# Non-Nucleoside Inhibitors Decrease Foot-and-Mouth Disease Virus Replication by Blocking the Viral 3D^pol^

**DOI:** 10.3390/v15010124

**Published:** 2022-12-30

**Authors:** Sirin Theerawatanasirikul, Ploypailin Semkum, Varanya Lueangaramkul, Penpitcha Chankeeree, Nattarat Thangthamniyom, Porntippa Lekcharoensuk

**Affiliations:** 1 Department of Anatomy, Faculty of Veterinary Medicine, Kasetsart University, Bangkok 10900, Thailand; 2 Department of Microbiology and Immunology, Faculty of Veterinary Medicine, Kasetsart University, Bangkok 10900, Thailand; 3 Center for Advanced Studies in Agriculture and Food, Kasetsart University Institute for Advanced Studies, Kasetsart University, Bangkok 10900, Thailand

**Keywords:** foot-and-mouth disease virus (FMDV), RNA-dependent RNA polymerase (RdRp), 3D polymerase (3D^pol^), small molecule, virtual screening, antiviral cell-based assay, antiviral agent

## Abstract

Foot-and-mouth disease virus (FMDV), an economically important pathogen of cloven-hoofed livestock, is a positive-sense, single-stranded RNA virus classified in the *Picornaviridae* family. RNA-dependent RNA polymerase (RdRp) of RNA viruses is highly conserved. Compounds that bind to the RdRp active site can block viral replication. Herein, we combined double virtual screenings and cell-based antiviral approaches to screen and identify potential inhibitors targeting FMDV RdRp (3D^pol^). From 5596 compounds, the blind- followed by focus-docking filtered 21 candidates fitting in the 3D^pol^ active sites. Using the BHK-21 cell-based assay, we found that four compounds—NSC217697 (quinoline), NSC670283 (spiro compound), NSC292567 (nigericin), and NSC65850—demonstrated dose-dependent antiviral actions in vitro with the EC50 ranging from 0.78 to 3.49 µM. These compounds could significantly block FMDV 3D^pol^ activity in the cell-based 3D^pol^ inhibition assay with small IC50 values ranging from 0.8 nM to 0.22 µM without an effect on FMDV’s main protease, 3C^pro^. The 3D^pol^ inhibition activities of the compounds were consistent with the decreased viral load and negative-stranded RNA production in a dose-dependent manner. Conclusively, we have identified potential FMDV 3D^pol^ inhibitors that bound within the enzyme active sites and blocked viral replication. These compounds might be beneficial for FMDV or other picornavirus treatment.

## 1. Introduction

Foot-and-mouth disease (FMD) is a highly contagious disease caused by the foot-and-mouth disease virus (FMDV). This disease is economically important and affects domestic livestock with cloven hooves worldwide [1]. FMD control relies on two major strategies: culling of infected animals and vaccination [2]. FMDV is a member of the *Picornaviridae* family, which consists of both animal and human pathogens, such as enterovirus, rhinovirus, and poliovirus. The FMDV genome is single-stranded RNA with positive polarity. Viral RNA synthesis during replication and transcription is achieved by RNA-dependent RNA polymerase (RdRp) encoded by 3D, so-called 3D^pol^ [3]. The 3D^pol^ of picornaviruses including FMDV and the RdRp of other RNA viruses share a common characteristic [4,5]. The three-dimensional structure of RdRp resembles a cupped right-handed configuration composed of three subdomains, the so-called thumb, fingers, and palm. The finger subdomain has extensions, termed fingertips, that bridge between the finger and the thumb subdomains to maintain the RdRp active site rearrangement and form the NTP entry site [6]. Therefore, the topology of these subdomains is essential for RdRp’s catalytic activity during negative-stranded RNA synthesis by holding the RNA template in place, engaging NTPs, and promoting polymerization. The central region of the 3D^pol^ contains six conserved motifs labelled A, B, C, D, E, and F, which are located mostly in the palm subdomain and form the template-binding channel with the 3D^pol^ active site [6]. Inhibition of 3D^pol^ activity can prevent viral genome replication. Therefore, because of its critical role in viral replication, 3D^pol^ is an attractive target for antiviral drug development.

As FMDV is highly contagious, broad-spectrum antiviral drugs as well as other novel natural and synthesized compounds might support the outbreak control by reducing spillover of the spreading virus into the environment. For example, the nucleoside analogue ribavirin (1-β-d-ribofuranosyl-1,2,4-triazole-3-carboxamide) is an antiviral drug licensed for the treatment of human and animal viral infections. However, ribavirin resistance caused by mutations within the RdRp coding sequences have been reported in many RNA viruses. For instance, poliovirus (PV) has substitution mutations, G64S and L420A, in 3D^pol^ while M296I was found in the FMDV 3D^pol^ [7,8]. Furthermore, a human hepatitis C virus (HCV) escape mutant contained the mutation Y415 in the NS5B gene [9]. M296 of FMDV 3D^pol^ is in the NTP binding site. Thus, alteration at this position may affect nucleotide recognition by the 3D^pol^ [10]. Previously, nucleoside analogues including favipiravir (T-705), T-1105 (3-oxo-3,4-dihydro-2-pyrazinecarboxamide derivative), and T-1106 were tested for their ability to inhibit FMDV infection in vitro and in vivo [11]. T-1105 demonstrated greater potency than T-705 and T-1106 to inhibit FMDV replication [11,12]. In an experimental FMDV infection, a high dose of T-1105 at 200 mg/kg twice daily for seven consecutive days was required to prevent FMD clinical signs and reduce viral shedding [12]. In a guinea pig model, the prophylactic effect of T-1105 was achieved at a high dose of 400 mg/kg/day twice daily for five consecutive days to give a similar protective level as the full-dose vaccination [13]. Thus far, no antiviral agent has been approved for the treatment or prevention of FMDV infection.

Among antiviral agents, nucleoside-based inhibitors (NIs) are likely to exhibit off-target effects and reduction in their potency in clinical cases [14]. Small molecules that target surface cavities or allosteric site of enzymes, so-called non-nucleoside inhibitors (NNIs), can be either natural or non-natural compounds [15]. Their structures are more flexible and thus can fit in the nucleotide-binding pocket better than the NIs [16]. Several NNI compounds have been reported to inhibit the RdRp activities of RNA viruses such as GPC-N114 in picornaviruses [14], lycorine in MERS-CoV [17], and NITD29 in Zika virus [18]. In addition to RdRp, 3C proteases (3C^pro^) are another attractive target for antiviral drug design as it is present and conserved across several positive-sense, single-stranded RNA viruses with picornavirus-like 3C^pro^ supercluster. It has been shown previously that three dipeptidyl-based synthetic molecules with different interactive chemical groups including an aldehyde (GC373), a bisulfite adduct (GC376), and an α-ketoamide (GC375) possessed broad-spectrum, potent antiviral effects [19]. These compounds effectively inhibited protease (3C^pro^ or 3CL^pro^) activities and replication of viruses from the families *Caliciviridae*, *Coronaviridae*, and *Picornaviridae*, as determined using FRET protease assay and cell-based assays, respectively.

In this study, we screened small molecules that targeted FMDV 3D^pol^ by integrating computer-aided virtual screening and a cell-based antiviral assay to filter for potential antiviral compounds. We further determined the inhibitory activity of the selected compounds on FMDV 3D^pol^ using a recently established FMDV minigenome with the green fluorescence reporter protein in the cell-based 3D^pol^ inhibition assay [20]. Taken together, we have identified four 3D^pol^ inhibitors and demonstrated the mechanism by which the small molecules could inhibit viral replication in FMDV-infected BHK-21 cells.

## 2. Materials and Methods

### 2.1. Virtual Screening of Small Molecules

The crystal structure of FMDV 3D^pol^ (FMDV RdRp) deposited in the PDB under a code 1wne.pdb was retrieved (https://www.rcsb.org/structure/1WNE). Upon the 3D structure’s preparation for the virtual screening, we found that four amino acids (F34, A68, E144, and K148) on the protein structures differed from the deduced amino acid sequence of FMDV O189. Then, homology modeling based on the 3D^pol^ crystal structure (PDB: 1wne.pdb) was performed on the SWISS-MODEL server (https://swissmodel.expasy.org/, accessed on 19 April 2021 [20]) to prepare the complete protein structure of O189 3D^pol^ previously used for the plasmid construction [20]. The structure quality was evaluated using MolProbity [21], Q-MEAN [22], and Ramachanadran plotting [23], as described elsewhere [20]. The FMDV 3D^pol^ model was prepared for the downstream process with the removal of ligands and water molecules and addition of hydrogen atoms. Molecular docking was achieved using the PyRx 0.9.8 virtual screening tool [24].

The compound libraries provided by the Developmental Therapeutics Program (DTP) Open Repository of the National Cancer Institute (NCI) were queried for a subset of the NCI repository collection comprising the NCI Diversity Set III, VI and V, and MechDiv3 libraries containing 5596 freely available models of compounds. Each subset of the compound models was assigned for docking using the Open Babel software toolbox [25], including Gasteiger partial charges, addition of hydrogen atoms, optimization of hydrogen bonds, and removal of atomic clashes, to generate the pdbqt input format.

Virtual screening was performed using AutoDock Vina [26] embedded in the PyRx 0.9.8 virtual screening tool [24]. In the first virtual screening, the small molecules retrieved from the model libraries were blind-docked onto the 3D^pol^ structure, which yielded a set of ligands predicted to bind the 3D^pol^ active sites or the conserved amino acid residues important for 3D^pol^’s function. Initially, the grid box was set at 65:70:65 (x, y, z dimensions in Angstroms), and the box was centered at 19.34:31.20:23.25 (x, y, z). The virtual screening outputs were presented as the predicted free energy in kcal/mol and the exhaustiveness value for the docking was set to 20. Subsequently, the top-ranked complexes were retrieved for the latter focus-docking. In the focus-docking, the top-ranked compounds with a binding energy lower than −8.0 kcal/mol were included, and the dockings were centered at specific sites or amino acid residues within the FMDV 3D^pol^. Three sites on the palm subdomain of FMDV 3D^pol^ involved in the nucleotide binding and polymerizations [6] were selected for the focus-docking. According to the 3D structure of FMDV 3D^pol^, site 1 is composed of D240, Y241, and D245 of Motif A in the loop β8-α9; site 2 comprises M296, S298, G299, S301, and N307 of Motif B in the loop β9-α11; and site 3 contains Y336, D338, and D339 of Motif C in the loop β10-β11. The grid box of 25:25:25 was assigned to these specific sites and centered at 15.50:26.22:15.00 (x, y, z). The promising docking results were harvested for protein–ligand visualization using Discovery Studio Visualizer, version 2021 (BIOVIA, Dassault Systèmes, San Diego, CA, USA) and UCSF Chimera, version 1.16 (UCSF, San Francisco, CA, USA).

### 2.2. Sources and Preparations of Chemicals

The freely available compounds were provided by the Developmental Therapeutics Program (DTP) Open Repository of the National Cancer Institute (NCI). Ribavirin (Sigma-Aldrich, St. Louis, MO, USA) and rupintrivir (Sigma-Aldrich, St. Louis, MO, US) were used as RdRp and non-RdRp inhibitor controls. All compounds were prepared as 10-mM stock solutions in 100% dimethyl sulfoxide (DMSO) (Sigma-Aldrich, St. Louis, MO, USA), and stored at −20 °C for the following in vitro cell-based experiments.

### 2.3. Cells and Viruses

Baby Hamster Kidney (BHK−21) cells and HEK-293T cells were obtained from American Type Culture Collection (ATCC^®^, Manassas, VA, USA). The cells were maintained in a complete medium containing Minimum Essential Medium (MEM, Invitrogen^TM^, Carlsbad, CA, USA), 10% fetal bovine serum (FBS, Invitrogen^TM^, Carlsbad, CA, USA), 2 mM L-glutamine (Invitrogen^TM^, Carlsbad, CA, USA), and 1×Antibiotic-Antimycotic (Invitrogen^TM^, Carlsbad, CA, USA) at 37 °C with 5% CO_2_. FMDV serotype A (NP05) was propagated in BHK21 cells at 37 °C with 5% CO_2_ for 24 h. The virus stock with a titer of 1 × 10^9^ TCID50/mL was stored at −80 °C in single-use aliquots. All works involving live FMDV were performed at biosafety level-2 with an enhanced facility.

### 2.4. Cytotoxicity Assay

BHK-21 cells were seeded at 1.8 × 10^4^ cells per well into 96-well plates (Corning Incorporated., Corning, NY, USA) and incubated at 37 °C with 5% CO_2_ overnight. The spent media was removed, and the cells were washed twice with 1× PBS. The compounds were serially diluted in serum-free MEM media containing DMSO at a final concentration of ≤0.1%. The diluted compounds were incubated with the cells or virus and the cells were further incubated at 37 °C for an additional 24 h. Two independent experiments were performed for all biological assays. Cytotoxicity was determined by measuring cell viability using MTS solution provided in the CellTiter 96^®^ Aqueous One Solution Cell Proliferation Assay (Promega, Madison, WI, USA) according to the manufacturer’s instructions. The absorbance of the solution in the experiment plates was measured at a wavelength of 490 nm using a multi-mode reader (Synergy H1 Hybrid Multi-Mode Reader, BioTek^®^, Winooski, VT, USA). Cell viability was calculated using the following formula (1), where OD_treate_ denotes the absorbance of the uninfected BHK-21 cell wells containing cells, media, and compounds; OD_cell control_ denotes the absorbance of the cell control wells containing cells and media; and OD_dmso_ denotes the absorbance of the vehicle control wells containing cells, media, and 0.1% DMSO.

[OD_treate_ − OD_cell control_]/[OD_dmso_ − OD_cell control_] × 100 (1)

### 2.5. Antiviral Activity Assay

The compounds that targeted FMDV 3D^pol^ in the virtual screening and could inhibit viral replication were further investigated for the effects on the other processes of viral infection as mentioned in the previous study [27]. Initially, we investigated the effects of compounds on viral entry including binding and penetration (pre-viral-entry experiment). The compound dilutions and FMDV at 10 TCID50 per wells were co-adsorbed onto the overnight-grown BHK-21 cells in a 96-well plate (Corning Incorporated., Corning, NY, USA) at 37 °C for 2 h. All virus–compound mixtures were removed, and the cells were washed twice with 1× PBS. Subsequently, fresh media was added to the cells which were further cultured at 37 °C for an additional 22 h. Secondly, in the post-viral-entry experiment, the BHK-21 cells in the 96-well plate were incubated with 10 TCID50 of FMDV per well at 37 °C for 2 h. Then, the cells were washed twice with 1× PBS, before treatment with the same compound dilutions that were used in the cytotoxicity assay at 37 °C for an additional 22 h to inhibit viral replication.

### 2.6. Immunoperoxidase Monolayer Assay (IPMA)

Immunoperoxidase monolayer assays (IPMA) were conducted as previously described [27,28]. Briefly, the BHK-21 cells, including no infection with and without compound and FMDV infection with and without compound, were fixed with cold methanol at room temperature for 20 min and washed with PBS plus 0.1% Tween 20 (1× PBST, Sigma Aldrich^®^, St. Louis, MO, USA). The cells were then incubated with single-chain variable fragment with Fc fusion protein (scFv-Fc) specific to 3ABC of FMDV [29] at 37 °C for 1 h for viral detection. After primary antibody incubation, the cells were washed with 1× PBST, and subsequently incubated with the protein G, HRP conjugate (dilution 1:1000, EMD Millipore corporation, Temecula, CA, USA) at 37 °C for 1 h. To visualize the antigen–antibody complex, the cells were stained with DAB substrate (DAKO, Santa Clara, CA, USA), and the dark-brown color of the infected FMDV cells was observed using a phase-contrast inverted microscope (Olympus IX73, Tokyo, Japan). The cell images were analyzed using CellProfiler image analysis v.4.2.0 [29]. The resulting data were used to calculate the half-maximal effective concentration (EC50). The EC50 value represented the compound concentration at which the virus infection was reduced by 50% compared to the DMSO control (FMDV infection with 0.1% DMSO). To analyze the data, the DMSO control was set at 100% infection, and the EC50 value of each compound was calculated using GraphPad Software version 9.4.1 (Prism, San Diego, CA, USA). The Z’ factor value was analyzed using the following formula (2) to assess the assay performances both within a plate and across plates.

Z’ factor = 1 − (3 × SD of cell control + 3 × SD of the virus control)/(mean cell control signal − mean virus control signal)(2)

A Z’ factor between 0.5 and 1 indicates an acceptable range.

### 2.7. Real-Time RT-PCR Assay

The whole viral RNA and negative-strand specific RNA of FMDV were detected and quantitated using real-time RT-PCR after treatment with the compounds. The BHK-21 cells were seeded at 1.8 × 10^5^ cells into each well of 24-well plates (Corning Incorporated., Corning, NY, USA) and cultured overnight. The cells were incubated with 10 TCID50 of FMDV for 2 h, and the inoculum was removed after viral adsorption. Then, the FMDV-infected cells were treated with serially diluted compounds at 37 °C as mentioned above. At 24 h post viral infection, intra- and extracellular viral RNA was isolated using Direct-zol^TM^ RNA MiniPrep (Zymo Research Corporation, Tustin, CA, USA) following the manufacturer’s instruction. The RNA was quantified using a NanoDrop^TM^ 2000c Spectrophotometer (Thermo Fisher Scientific, Waltham, MA, USA) and used as the templates for cDNA synthesis. The first-strand cDNAs derived from the whole viral RNA were generated using Random hexamers (Invitrogen^TM^, Carlsbad, CA, USA) for whole viral RNA quantification. On the other hand, a primer specific to the negative-stranded RNA within the 3D^pol^ coding sequence (5′-AAGGGTTGATTGTTGACA-3′) was employed to amplify the first-strand cDNA for the negative-stranded RNA quantification [30]. Both cDNA syntheses were performed with the enzyme SuperScript III Reverse Transcriptase (Invitrogen^TM^, Carlsbad, CA, USA) according to the manufacturer’s instructions.

The cDNA templates were subjected to downstream DNA quantification with qPCR using iTaq Universal SYBR Green Supermix (Bio-Rad Laboratories, Hercules, CA, USA). The primer sets for the DNA amplifications are listed in Table 1. Briefly, 5 µL mixture containing 0.5 µL of each target-specific forward and reverse primer was mixed with 2 µL cDNA and nuclease-free ddH_2_O up to a final volume of 10 µL. The qPCR amplifications were conducted in a two-step method: first, denaturation at 95 °C for 30 sec, followed by 40 cycles of denaturation at 95 °C for 5 sec and annealing/extension at 55 °C for 30 sec. A melting curve was analyzed from 65 °C to 95 °C with 0.5 °C increments using a CFX96 touch Real-Time PCR detection system (Bio-Rad Laboratories, Hercules, CA, USA).

Viral RNA expression was determined using the absolute quantification method [27]. A standard curve of DNA derived from the whole viral RNA was generated from the ten-fold serially diluted plasmid containing FMDV 5′UTR from 10^−2^ to 10^−7^ plasmid molecules/µL. Copy numbers of the DNA were calculated based on the standard curve. The negative-stranded RNA is normally produced by 3D^pol^ during viral replication. The negative-stranded RNA-derived DNA was quantitated based on the delta Ct values (cycle threshold) by subtracting the Ct values of the virus samples (FMDV-infected BHK-21 cells with compound treatment) from the Ct values of the virus control (FMDV-infected BHK-21 cells without compound treatment). The data were normalized using the following Equation (3).

Normalized delta Ct = 1 − (delta Ct of sample)/(Ct of dmso)(3)

The normalized data from three independent replications are presented as the means ± SD using GraphPad Prism version 9.4.1 (Prism, San Diego, CA, USA).

### 2.8. Cell-Based 3D^pol^ Inhibition Assay

Inhibitory effects of the compounds on FMDV 3D^pol^ were examined using plasmid pKLS3_GFP, an FMDV minigenome expressing GFP, and the two helper plasmids pCAGGS_T7 and pCAGGS_P3 [20]. pKLS3_GFP contains the enhanced green fluorescent protein (GFP) gene inserted between FMDV O189 5′ and 3′UTRs required for FMDV transcription and translation, while pCAGGS_T7 and pCAGGS_P3 are mammalian protein expression plasmids containing T7 RNA polymerase and the FMDV P3 region essential for efficient generation of FMDV RNA, respectively. 

The three plasmids were transfected onto the BHK-21 cells as described previously [20]. Briefly, BHK-21 cells were seeded at 1 × 10^4^ cells/well into 96-well plates and incubated at 37 °C overnight. On the transfection day, 40 ng of pKLS3_GFP, 120 ng of pCAGGS_T7, and 40 ng of pCAGGS_P3 were mixed with 0.6 µL of Fugene^®^ HD (Promega, Madison, WI, USA) in Opti-MEM™ I Reduced-Serum Medium (Thermo Fisher Scientific, Waltham, MA, USA) to make a final volume of 50 µL per well. Then, the spent media was removed and replaced with the transfection mixture and incubated at 37 °C for 4 h. Subsequently, the transfection mixture was removed and replaced with the serially diluted compounds in Opti-MEM^TM^ I Reduced-Serum Medium (Gibco^TM^ Thermo Fisher Scientific Inc., Waltham, MA, USA). In the vehicle control well, 0.1% DMSO was added in the wells instead of the compounds and it also served as the transfection positive control. pKLS3_GFP without helper plasmid transfection was also included as the plasmid control. The cells were incubated at 37 °C with 5% CO_2_ and the fluorescent reporter expression signals were observed at 24–48 h post transfection using a phase-contrast inverted microscope (Olympus IX73, Tokyo, Japan).

The levels of FMDV 3D^pol^ activity corresponded to the numbers and intensity of the bright green fluorescent signal in the positive cells. The background was adjusted for contrast and brightness only. The fluorescent intensities of the images were measured, and the background was subtracted from each image using CellProfiler image analysis v.4.2.0 [29]. The half-maximal inhibitory concentration (IC50) is the concentration at which the 3D^pol^ activity was reduced by 50% compared to that of the DMSO control. For the analysis, the signal of the DMSO control was set at 100% and the IC50 was calculated using GraphPad Software version 9.4.1 (Prism, San Diego, CA, USA).

### 2.9. Cell-Based FMDV 3C^pro^ Inhibition Assay

3C^pro^ is the main protease crucial for FMDV biology and is an attractive antiviral target. Therefore, we were interested to know whether these compounds could inhibit the 3C^pro^ activity. We determined the effects of the compounds on the main protease using a cell-based FMDV 3C^pro^ inhibition assay as described previously [27,31]. Briefly, HEK-293T cells were grown in 96-well plates at 1 × 10^3^ cells per well. The cells were maintained in Opti-MEM I Reduced-Serum Medium (Gibco^TM^ Thermo Fisher Scientific Inc., Waltham, MA, USA). On the next day, the cells were transfected with plasmids pG5Luc (Promega, Madison, WI, USA) and pBV_3ABCD expressing intact 3C^pro^ or pBV_mu3ABCD expressing inactive 3C^pro^ [32]. The 3ABCD and mu3ABCD genes were inserted between the Gal4-binding domain and the VP16-activation domain of plasmid pBV (Promega, Madison, WI, USA). pG5Luc is a reporter plasmid containing the GAL4 binding site upstream of VP16 and the firefly luciferase reporter gene sequences followed by the downstream *Renilla* luciferase gene. The total 10 µL of co-transfection mixtures comprising 0.1 µg pBV_3ABCD or pBV_mu3ABCD, 0.1 µg pG5Luc, and 0.6 µL Fugene^®^ HD (Promega, Madison, WI, USA) were incubated with HEK-293 T cells at 37 °C for 2 h before adding the diluted compounds. At 16 h post transfection, the transfected cells were lysed with 20 µL passive lysis buffer (Promega, Madison, WI, USA) followed by firefly and *Renilla* luminescence signal determination using the Dual-Glo Luciferase Assay System (Promega, Madison, WI, USA) in a Synergy H1 Hybrid Multi-Mode Microplate Reader (BioTek, Winooski, VT, USA). The intact 3C^pro^ expressed from pBV_3ABCD would separate the Gal4-binding domain from the VP16-activation domain, resulting in no expression of the firefly luciferase signal. When the compounds could suppress the 3C^pro^, the Gal4-binding domain and the VP16-activation domain were in close proximity. Thus, GAL4 bound to the Gal4-binding domain and drove the firefly and *Renilla* luciferase expressions. The data were recorded as an inverse correlation of the firefly/*Renilla* luminescent (Fluc/Rluc) signal ratio from compound-treated wells compared to those obtained from wells transfected with pBV_mu3ABCD (3C^pro^ negative control) and pBV16 (empty plasmid control).

## 3. Results

### 3.1. Virtual Screening of Small Molecules

**Figure 1:**
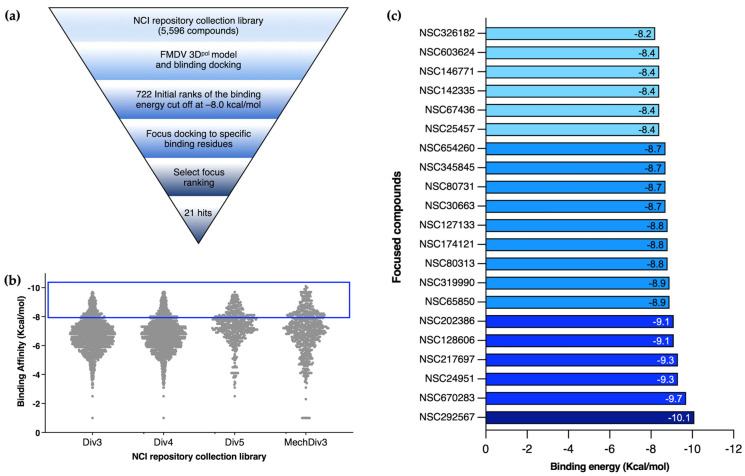
Virtual screening approach in this study. Schematic diagram of virtual screening starting from the initial docking to focus-docking (**a**). The predicted binding affinities of all small molecules, from which the cut-off was set at −8.0 kcal/mol to filter for the second molecular screening (**b**). The twenty-one top-ranking focal compounds chosen for further cell-based experiments (**c**).

We screened compounds from the model libraries to identify novel inhibitors specific to FMDV 3D^pol^ (Figure 1). In the initial in silico virtual screening of 5596 NCI compounds, the binding energy ranged from −1.5 to −10.1 kcal/mol. We then used a cut-off of −8.0 kcal/mol to select the 722 compounds that occupied the enzyme active sites of FMDV 3D^pol^ for further focus-docking. The focus-docking was narrowed down to the specific amino acid residues within the three active sites of the FMDV 3D^pol^ structure: site 1: D240, Y241, and D245 of Motif A; site 2: M296, S298, G299, S301, and N307 of Motif B; and site 3: Y336, D338, and D339 of Motif C. Upon the blind- and focus-virtual screenings, the 21 top-ranked compounds were selected for the following experimental validation. The binding affinities of the 21 compounds are listed in Figure 1c.

### 3.2. Dose–Response Analysis on the Cell-Based Antiviral Activity of Small Molecules

First, we tested the cytotoxicity and antiviral activities of the 21 focal compounds in BHK-21 cells. The cytotoxicity profile of the small compounds in BHK-21 cells was determined by measuring the MTS formazan product of living cells. The four compounds that were not toxic to the BHK-21 cells and had CC50 values in the mid to high micromolar range (49.91 ± 1.70 µM to >100 µM) were tested in a dose–response manner. Therefore, the highest non-toxic concentration of each compound was used in the subsequent antiviral activity assay. Among the 21 compounds, NSC217697, NSC670283, NSC292567, and NSC65850 demonstrated antiviral inhibition at the maximum dose of non-toxic concentration under the post-viral infection condition using 10 TCID50 of FMDV and observing at 24 hpi.

To validate these findings in a dose-dependent manner, we investigated the antiviral properties of the four compounds in FMDV-infected BHK-21 cells. In the pre-viral-entry experiment, unabsorbed FMDV were removed, and the cells were washed with PBS before adding the fresh media. The four compounds also showed viral inhibition during co-absorption with the virus on the cell monolayer for 2 h (in Figure S1). High concentrations of the compounds were required to exert the viral-suppression effects compared to the same compound examined in the post-viral-entry assay. We assumed that the compounds still had sufficient potency to reduce FMDV after being removed and washed away.

**Figure 2:**
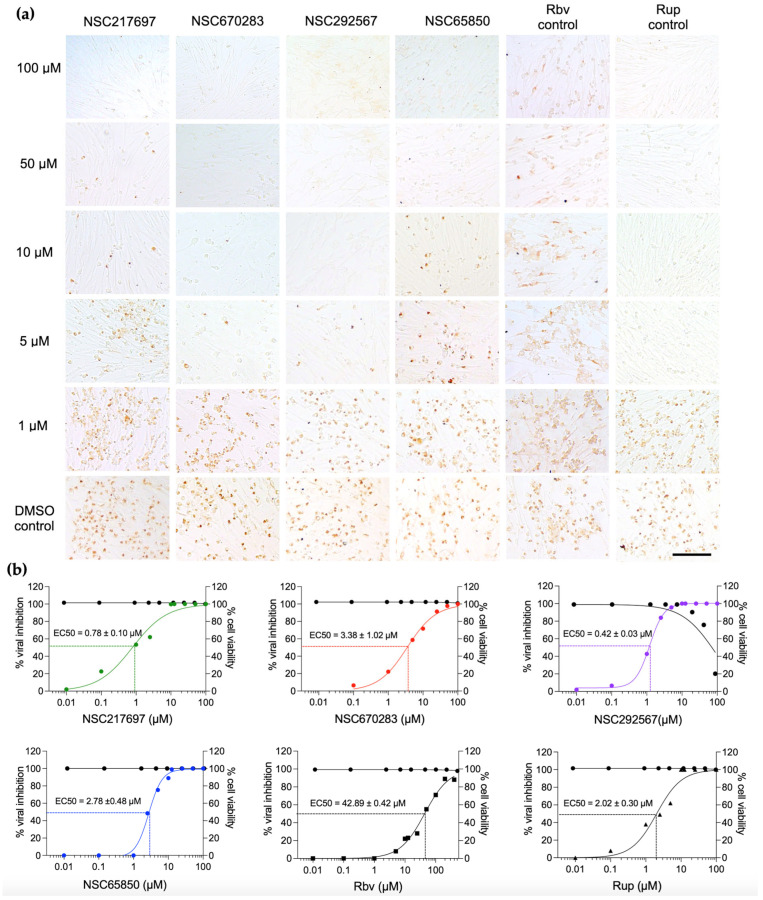
Antiviral activities of the selected compounds against FMDV in cell culture. Four small compounds were evaluated in a dose–response manner, and ribavirin (Rbv) and rupintrivir (Rup) were used as drug controls. The FMDV-infected cells with or without compound treatment were determined using IPMA in which viral antigens in the positive cells were stained with DAB and turned brown (**a**). Graphs of cytotoxicity and antiviral activities of the compounds demonstrating percentage of viable cells (black lines) and percentage of viral inhibition (color lines) at different concentrations of the compounds (**b**). The scale bar is 100 µm.

We next determined whether the candidate compounds had antiviral activity during post-viral infection by infecting BHK-21 cells with FMDV at 10 TCID50, followed by incubating with serial concentrations of each compound. The results showed that the numbers of positive infected cells were markedly decreased with the increased compound concentrations in a dose-dependent manner as shown in Figure 2. The vehicle control (0.1% DMSO) did not influence viral production in the infected cells. NSC217697 and NSC292567 exhibited great antiviral inhibition of 0.78 ± 0.10 and 0.42 ± 0.08 µM, respectively. NSC670283 and NSC65850 demonstrated good inhibitory effects with the EC50 of 3.38 ± 1.02 and 2.73 ± 0.48 µM, respectively (Table 2). These four compounds showed antiviral activities greater than ribavirin (the broad-spectrum anti-RdRp nucleoside analogue) and had antiviral potency comparable to rupintrivir (the anti-3C^pro^ peptidomimetic drug of human rhinovirus) as shown in Figure 2. In addition, the off-target compounds, which did not bind to the 3D^pol^ active site, or bound with the higher binding affinities (>−8.0 kcal/mol), were also examined for cytotoxicity using MTS and antiviral activities in the cell-based assay with immunostaining by IPMA. The results showed that the random or non-target compounds did not inhibit viral replication in both pre- and post-viral entry as shown in Figure S2.

Furthermore, the EC50 and EC90 values, which are concentrations of compounds that could reduce 50% and 90% of the viral infectivity, respectively, were determined in the cell-based assay and IPMA. Both values were calculated using non-linear regression to determine the SI (selective index) by CC50/EC50. The SIs of the four compounds on suppression of FMDV infection in BHK-21 cells are shown in Table 2. NSC217697 and NSC292567 showed SI values >100.

### 3.3. Functional Interference on FMDV 3D^pol^, but Not 3C^pro^, by the Candidate Compounds

**Figure 3:**
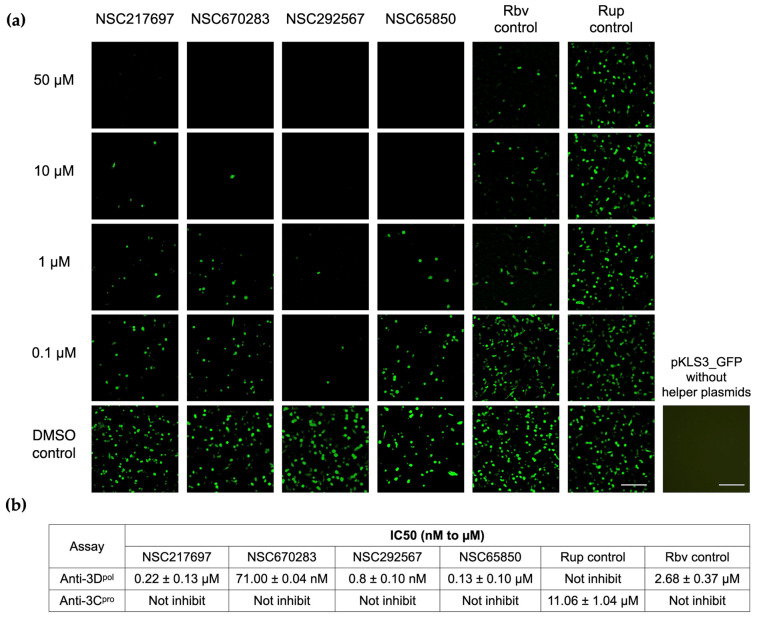
Cell-based 3D^pol^ inhibition assay based on FMDV minigenome expressing green fluorescent protein. 293T cells were transfected with pKLS3_GFP and two helper plasmids, and then treated with the selected compounds in a dose-dependent manner. Ribavirin (Rbv) and rupintrivir (Rup) were used as 3D^pol^ positive and negative drug controls, respectively, and 0.1% DMSO served as a vehicle control. No fluorescent signal was observed in the transfection well containing pKLS3_GFP in the absence of the two helper plasmids (plasmid control). The bars are 100 µM (**a**). The table shows 50% inhibitory concentration (IC50) values of the four compounds, ribavirin (Rbv), and rupintrivir (Rup) (**b**). Please note that ribavirin is the RdRp inhibitor and rupintrivir is a 3C^pro^ inhibitor.

We further investigated the mechanism by which the compounds inhibited FMDV 3D^pol^ activity with a cell-based 3D^pol^ inhibition assay exploiting pKLS3_GFP, an FMDV minigenome with the GFP gene. pKLS3_GFP contains essential elements of FMDV replication and the GFP reporter gene while one of the helper plasmids provides functional FMDV 3D^pol^. The numbers of GFP-positive cells and their intensity reflect the 3D^pol^ activity. Antiviral replication of the compounds on FMDV 3D^pol^ was measured as the decreased GFP intensity and positive cell numbers in the compound treatment well related to those in the vehicle control well (Figure 3a). The results showed that NSC670283 and NSC292567 could inhibit FMDV 3D^pol^ activity in nanomolar concentrations with the IC50 equal to 71.00 ± 0.04 nM and 0.8 ± 0.10 nM, respectively, while NSC217697 and NSC65850 also demonstrated good inhibitions at low micromolar concentrations of 0.22 ± 0.13 µM and 0.13 ± 0.10 µM, respectively (Figure 3a and 3b). All candidate compounds showed inhibitory effects greater than ribavirin (IC50 = 2.68 ± 0.37 µM). We also sought to know the inhibitory effects of the compounds on other viral proteins, because 3C^pro^ plays a major role in picornavirus polyprotein maturation, essential in the viral life cycle. We further investigated this function by testing the compounds using the cell-based FMDV 3C^pro^ inhibition assay. The results demonstrated that none of the candidate compounds showed an effective inhibitory effect on FMDV 3C^pro^. Therefore, these data highlighted the anti-3D^pol^ activity of the four candidate compounds (Figure 3).

### 3.4. Inhibition of FMDV Replication by Small Compounds

**Figure 4:**
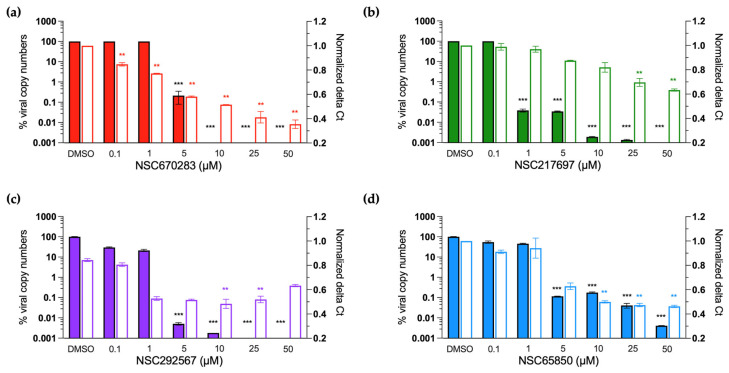
Percent viral copy numbers and the delta Ct values of negative-stranded RNA of FMDV-infected BHK-21 cells with or without compound treatments. The infected cells were treated with serial concentrations of the selected compounds to demonstrate the dose-dependent effect. The selected compounds included NSC670283 (**a**; red), NSC217697 (**b**; green), NSC292567 (**c**; purple), and NSC65850 (**d**; blue). The bar graphs were plotted between differential drug concentrations (*X*-axis), % viral copy number (left *Y*-axis), and the normalized delta Ct values of negative-stranded RNA (right *Y*-axis). The solid-color bars represent % viral copy numbers and the empty bars are the normalized delta Cts. The asterisks denote the significance levels with ** meaning a *p* value of 0.01 and *** a *p* value of 0.001.

We further examined the effects of the four compounds on the viral replication process by measuring levels of viral loads and negative-stranded RNA synthesis by FMDV 3D^pol^ using RT-qPCR (Figure 4). Firstly, we investigated the negative effects of the compounds on the negative-stranded RNA synthesis at 0.1, 1, 5, 10, 25, and 50 µM (Figure 4). The results showed that all four compounds could reduce the levels of negative-stranded RNA in a dose-dependent manner. Secondly, viral copy numbers derived from FMDV-infected BHK-21 cells treated with serial concentrations of the four compounds were determined to reveal their inhibition activity on viral loads. The results showed that all compounds had inhibition activities over 99% at 5 µM (Figure 4). Among the four compounds, NSC217697 appeared to be the most potent inhibitor with an inhibition ratio > 99% at 1 µM. The results showed that the compounds could actively suppress both viral load and negative-RNA strand production of 3D^pol^. These data confirmed that the four candidate compounds were potent FMDV 3D^pol^ inhibitors by inhibiting the viral RNA synthesis function of the enzyme.

### 3.5. The Predicted Interaction of the Small Compounds and the Catalytic Active Sites of FMDV 3D^pol^

**Figure 5:**
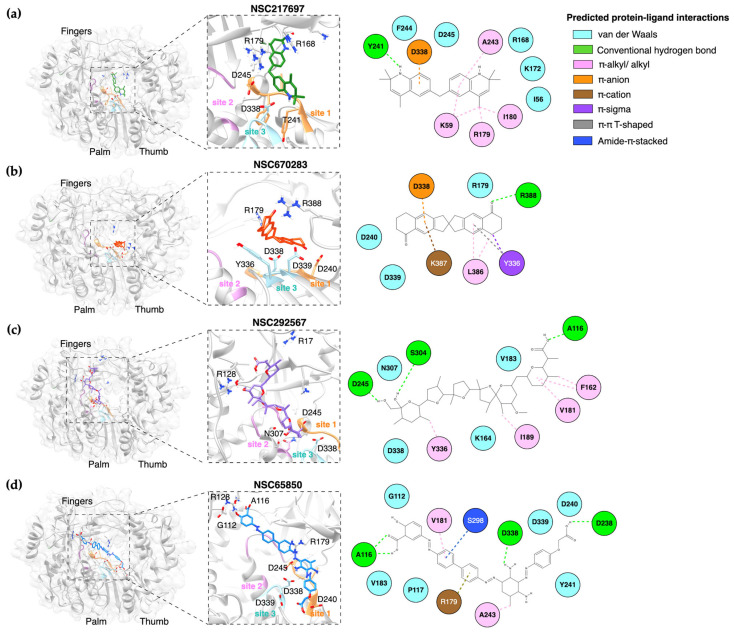
The predicted 3D (left panel) and 2D structures (right panel) of the small molecule–FMDV 3D^pol^ interactions. Interactions between the residues within sites 1–3 of FMDV 3D^pol^ and small compounds NSC217697 (**a**), NSC670283 (**b**), NSC292567 (**c**), and NSC65850 (**d**) are demonstrated.

The focused molecular docking showed that the four compounds occupied the 3D^pol^ active sites and they inhibited the 3D^pol^ function in the cell-based assay. We further investigated the protein–ligand interactions of the compounds docked to these sites within the 3D^pol^ palm and finger subdomains. The protein–ligand interactions are shown in Figure 5. All compounds properly located to site 1 of the loop β8-α9 and site 3 of the loop β10-β11 rather than site 2. The binding showed that NSC217697 formed a hydrogen bond with Y241 and van der Waals bond with D245 of the loop β8-α9 and reacted to D338 with π-anion. Moreover, this compound could bind to the NTP binding residues (R168, K172, and R179) of the finger subdomain with alkyl and van der Waals interactions (Figure 5a). As the catalytic aspartates and NTP binding site play an important role in RNA–3D^pol^ interaction, NSC217697 was predicted to bind and inhibit the interaction between RNA and 3D^pol^ molecule. NSC670283 is composed of fused bicyclic rings with cyclic ketone. D338 of motif C reacted to the bicyclic rings with π-anion interaction. Moreover, Y336 also shared the binding to cyclic ketone with π-sigma bond. The L386, K387, and R388 of motif E that are crucial for interacting with the RNA formed π-alkyl/alkyl and π-cation bonds to the bicyclic rings and H-bonds to the ketone group (Figure 5b). When the ionophore antibiotic bound to FMDV 3D^pol^, NSC292567 formed three hydrogen bonds with D245, S304, and A116. The Y336 (motif C) of the loop β10-β11 reacted to the compounds by alkyl interaction, and N307 (motif B) formed van der Waals interactions with NSC292567 (Figure 5c). NSC65850 occupied all three sites and formed van der Waals bonds with D240 and Y241 (site 1), amide-π stacked interactions with S298 (site 2), and hydrogen bonds with D338 (site 3) (Figure 5d). These predicted interactions showed that the candidate compounds could structurally interfere with the RNA polymerase activity of FMDV 3D^pol^.

## 4. Discussion

RNA-dependent RNA polymerase (RdRp) is the enzyme essential for replication and transcription processes of RNA viruses including FMDV and other picornaviruses. Therefore, RdRp is an attractive target for novel inhibitors against RNA viruses. In the case of FMDV, the crystal structures of RdRp (3D^pol^) have been resolved, and are similar to the right-handed architecture with three conserved subdomains—palm, finger, and thumb. This characteristic is common among picornaviruses [5,6]. Computational drug discovery has proven to be a guide for screening new drug candidates, and this method can directly provide scientific evidence for further in vitro and in vivo testing. In this study, we optimized the FMDV 3D^pol^ model for small-molecule screening processes. Initially, we performed the blind virtual screening of small molecules to filter the 3D^pol^ binding compounds for the second-round focused docking. The focused molecular docking specified the compounds that fit the active sites and involved functional areas of FMDV 3D^pol^. We demonstrated that four out of 21 compounds obtained from the double virtual screening inhibited FMDV at the post-infection stage using a cell-based antiviral assay. Moreover, these candidates showed good inhibition specific to FMDV 3D^pol^ activity, but not 3C^pro^. 3D^pol^ is one of the most important enzymes for viral replication processes and it functions after viral infection. In our study, ribavirin could inhibit FMDV 3D^pol^ as it is a well-known broad-spectrum anti-RNA virus and the complex of FMDV RdRp, RNA template, and ribavirin triphosphate has been solved and demonstrated [10]. In addition, a ribavirin-resistant mutant, which contained M296I on the β9-α11 loop adjacent to the active site, induced misincorporation of guanosine monophosphate into the RNA chain leading to an error catastrophe [8,10,33]. Therefore, it is suitable to be used as a positive RdRp inhibitor.

We have shown that the four compounds, NSC217697, NSC670283, NSC292567 and NSC65850, interacted with the active sites of FMDV 3D^pol^ to inhibit the enzyme function, and thus markedly reduced synthesis of viral RNA. NSC217697 (6,6′-methylene bis(2,2,4-trimethyl-1,2-dihydroquinoline)) is a quinoline, which contains fused rings of heterocyclic compounds. We found that this compound could inhibit FMDV replication after infection. Moreover, NSC217697 could inhibit 3D^pol^ with a micromolar concentration, but had no inhibitory effect on FMDV 3C^pro^. This compound was predicted to bind the NTP binding residues (R168, K172, and R179) in the finger subdomain, which possibly masked an initiation site in the complex conformation. Recently, quinoline and quinazoline derivatives have been shown to inhibit SARS-CoV-2 RdRp [34]. We assumed that NSC217697 could be an allosteric inhibitor of FMDV 3D^pol^.

NSC670283 (2,2′-spirobi [3,6,7,8-tetrahydro-1H-cyclopenta[g]naphthalene]-5,5′-dione) is a spiro compound that contains a fused unit of 1,2,3,6,7,8-hexahydro-5*H*-cyclopenta[*b*]naphthalen-5-one at position 2. This spiro compound has been reported to predictably interact with hepatitis C virus E2 envelope glycoprotein (HCV E2). It could bind HCV E2 protein as detected by surface plasmon resonance [35]. According to a viral specificity test, NSC670283 also inhibited RD114 glycoprotein (RD114pp) of endogenous feline retrovirus with IC50 of 3 µM in Huh-7 cells. However, antiviral activity against HCV replication in cell culture was not determined in the previous study. Indeed, we have shown that NSC670283 reacted to specific amino acid residues within Motifs C and E and the catalytic aspartic residue in the palm subdomain of FMDV 3D^pol^. This compound could inhibit FMDV replication as well as FMDV 3D^pol^ activity with micromolar IC50 and EC50, suggesting a potential broad-spectrum antiviral.

Among the four compounds, NSC292567 (nigericin or pandavir) demonstrated the highest binding affinity. This compound is grouped with the ionophore antibiotics, which have inhibitory activities against drug-resistant strains of Gram-positive bacteria and coccidian protozoa. It has been used to treat coccidiosis in poultry [36]. During the global COVID-19 crisis, the ionophore antibiotics were a group of interesting drugs that were repurposed for antiviral drug development. For example, monesin, a monovalent cation/proton antiporter, was found to inhibit MERS-CoV [37] and SARS-CoV-2 [38]. Salinomymin could inhibit influenza A and B viruses by blocking endosomal acidification and interfering with viral matrix protein 2 [39]. In addition, nigericin is an ionophore that affects lipid-soluble molecules. It increases the permeability of an ion across a biological membrane as well as the lipid bilayer of the vesicular transport system that functions in cellular trafficking of protein macromolecules. Picornavirus replication requires maturation of these membranous vesicles in the vesicular compartment during plus- and minus-strand RNA syntheses. Disruption of this membranous vesicle by ionophores thus inhibits viral replication [40]. Nigericin demonstrated a moderate inhibition of SARS-CoV-2 [38]. In this study, nigericin was ranked in the first place of our virtual screening with a binding affinity of −10.1 kcal/mol, and it had a good inhibitory effect on FMDV replication with an EC50 value of 0.42 µM. Moreover, nigericin at 0.8 nM reduced FMDV 3D^pol^ activity by 50%, and the anti-3D^pol^ action of this compound was confirmed by the reduction of negative-stranded RNA synthesis. In our predicted model study, nigericin could occupy the finger and palm subdomains of 3D^pol^. These results suggest that the ionophore antibiotics could be potential antivirals against FMDV infection by directly suppressing RNA synthesis of 3D^pol^.

NSC65850 (5-((4′-((2,4-diamino-3-((4-(carboxymethoxy)phenyl)diazenyl)-5-methylphenyl)diazenyl)[1,1′-biphenyl]-4-yl)diazenyl)-2-hydroxybenzoic acid) is a compound in the NCI diversity set III. Thus far, its antimicrobial effect has not been reported in the available databases. Therefore, we further investigated the physicochemical properties of this compound. The predicted model based on the SwissAMDE analysis [41] (accessed on 1 August 2022) demonstrated that this compound possessed a poor solubility (Log *S* = −11.83), Log *P*_o/w_ of 7.17, low skin permeation (log *K*_p_ = −5.14 cm/s), and low GI absorption. However, its synthetic accessibility score was 4.25, which is moderate for chemical modification in medicinal chemistry to improve its physicochemical property for druggable agents. Nonetheless, our experiments in the cell-based assay showed that this compound could inhibit 3D^pol^, but not 3C^pro^ of FMDV.

## 5. Conclusions

In conclusion, we have identified novel small-molecule inhibitors of foot-and-mouth disease virus 3D polymerase with virtual screening and further evaluated their actions in both virus-infected cells and a direct target to the polymerase protein. We provided evidence that NSC217697 (quinoline compound), NSC670283 (spiro compound), NSC292567 (ionophore antibiotic), and NSC65850 inhibited FMDV replication by binding to 3D^pol^ and suppressing viral RNA synthesis. Thus, these compounds can be considered as repurposing antiviral agents or as chemical scaffolds for FMDV and other picornavirus drug development.

## Figures and Tables

**Table 1:** Primers used in this study for amplification of FMDV cDNAs.

Primers	Sequence (5′–3)	cDNA Derived from	Tm (°C)	References
FMDV-5′UTRF	CTGTTGCTTCGTAGCGGAGC	5′UTR	66.3	[20]
FMDV-5′UTRR	TCGCGTGTTACCTCGGGGTACC		66.3	
FMDV-3DF	TAGAGCAGTAGATGTTG	Negative strand	58	In this study
FMDV-3DR	ATGAACATCATGTTTGAGG		59	

**Table 2:** The cell-based antiviral activity against FMDV infection and cytotoxicity of compounds in BHK-21 cells.

Compounds	Cytotoxicity CC50 (µM)	Antiviral Activity		SI (CC50/EC50)
		EC50 (µM)	EC90 (µM)	
NSC217697 6,6′-methylene bis(2,2,4-trimethyl-1,2-dihydroquinoline) PubChem ID 99342	>100	0.78 ± 0.10	9.52 ± 0.18	>128.20
NSC670283 2,2′-spirobi [3,6,7,8-tetrahydro-1H-cyclopenta[g]naphthalene]-5,5′-dione PubChem ID 382634	>100	3.38 ± 1.02	30.45 ± 0.25	>28.65
NSC292567 Pandavir/Nigericin PubChem ID 4490	49.91 ± 1.70	0.42 ± 0.08	3.85 ± 0.34	>118.83
NSC65850 sodium;5-[[4-[4-[[2,4-diamino-3-[[4-(carboxymethoxy)phenyl]diazenyl]-5-methylphenyl]diazenyl]phenyl]phenyl]diazenyl]-2-hydroxybenzoic acid PubChem ID 135422251	>100	2.73 ± 0.48	7.19 ± 0.10	>36.63
Ribavirin PubChem ID 37542	>100	42.89 ± 0.42	393.60 ± 2.56	>2.33
Rupintrivir PubChem ID 6440352	>100	2.02 ± 0.30	12.05 ± 1.6	>49.50

## Data Availability

No new data were created or analyzed in this study. Data are available upon request.

## Supplementary Materials

The following supporting information can be downloaded at: https://www.mdpi.com/article/10.3390/v15010124/s1, Figure S1: Pre-viral entry effect of the selected compounds (colored lines) and their cytotoxicity (black line). The inhibitory effects of the compounds on viral infection were presented by color line graphs plotted between % viral inhibition (left *Y*-axis) and various concentrations of each compound: NSC217697 (a; green), NSC670283 (b; red), NSC292567 (c; purple), and NSC65850 (d; blue). The EC50 value of each compound was also shown. The cytotoxicity of each compound was depicted as a black line graph by plotting differential doses of each compound (*X*-axis) against % cell viability (right *Y*-axis); Figure S2: Non-target compounds from the blind- and focus- molecular dockings. The compounds with the binding affinities higher than the cutoff of −8.0 kcal/mol or those that did not bind in the 3D^pol^ active site were examined for cytotoxicity using MTS (a). The interactions between the non-target compounds (green pointing with yellow arrow) and FMDV 3D^pol^ are demonstrated (b, left panel). The antiviral activities of the compounds at 50 μM were evaluated in the cell-based pre- and post-viral-entry assays and IPMA (b, right panel). The scale bar is 100 μm.
