# Supplementary material for: Non-Nucleoside Inhibitors Decrease Foot-and-Mouth Disease Virus Replication by Blocking the Viral 3Dpol
Source: Viruses. 2022 Dec 30;15(1):124. doi: 10.3390/v15010124 (PMC9866314; doi:10.3390/v15010124)
Supplement: Supplementary file 1 [file viruses-15-00124-s001.zip › viruses-2066399-supplementary.pdf]

Supplementary data

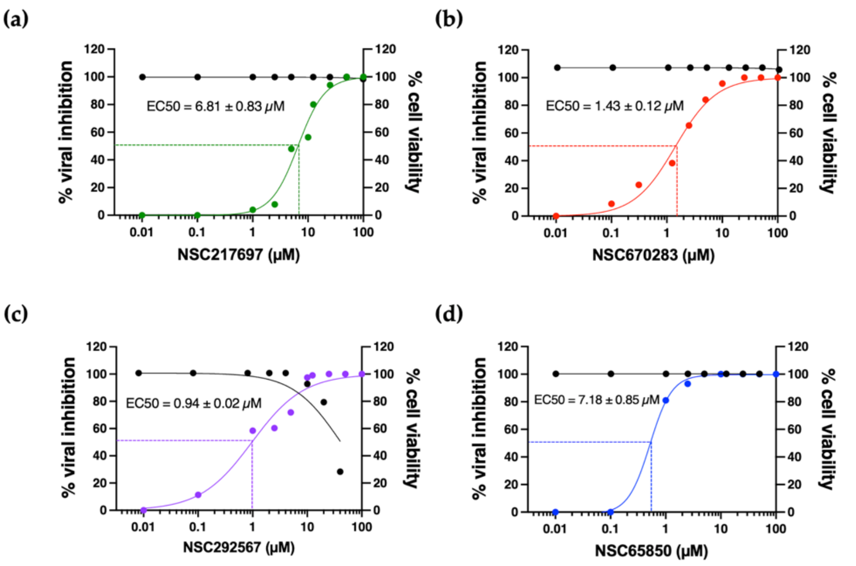

Figure S1. Pre-viral entry effect of the selected compounds (colored lines) and their cytotoxicity (black line). The inhibitory effects of the compounds on viral infection were presented by color line graphs plotted between % viral inhibition (left Y-axis) and various concentrations of each compound: NSC217697 (a; green), NSC670283 (b; red), NSC292567 (c; purple), and NSC65850 (d; blue). The EC50 value of each compound was also shown. The cytotoxicity of each compound was depicted as a black line graph by plotting differential doses of each compound (X-axis) against % cell viability (right Y-axis).

(a)

Non-target compounds	CC50 (μM)
NSC64672	956.00
NSC94600	678.80
NSC326184	806.60
NSC623059	503.10
NSC320218	175.20

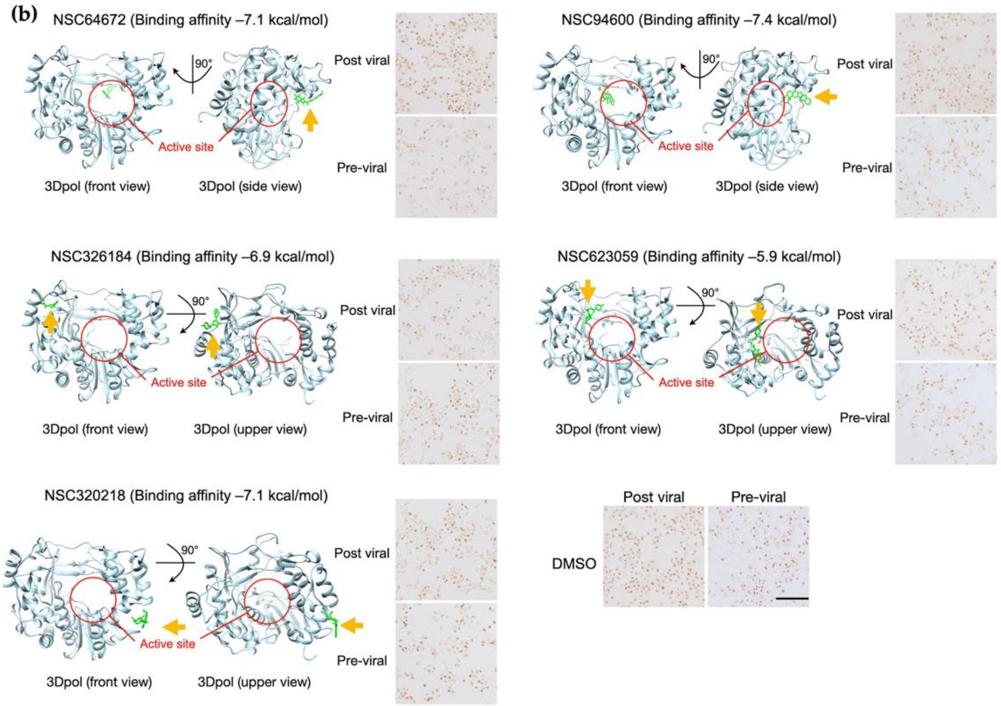

Figure S2. Non-target compounds from the blind- and focus- molecular dockings. The compounds with the binding affinities higher than the cutoff of –8.0 kcal/mol or those that did not bind in the 3Dpol active site were examined for cytotoxicity using MTS (a). The interactions between the non-target compounds and FMDV 3Dpol (green) are demonstrated (b, left panel). The antiviral activities of the compounds at 50 μM were evaluated in the cell-based pre- and post- viral entry assays and IPMA (b, right panel). The scale bar is 100 μm.
